# Porous TiO_2_-Based Gas Sensors for Cyber Chemical Systems to Provide Security and Medical Diagnosis

**DOI:** 10.3390/s17122947

**Published:** 2017-12-19

**Authors:** Vardan Galstyan

**Affiliations:** Sensor Lab, Department of Information Engineering, University of Brescia, Via Valotti 9, 25133 Brescia, Italy; vardan.galstyan@unibs.it; Tel.: +39-030-371-5702

**Keywords:** TiO_2_, porous structure, tubular structure, gas sensor, cyber chemical system

## Abstract

Gas sensors play an important role in our life, providing control and security of technical processes, environment, transportation and healthcare. Consequently, the development of high performance gas sensor devices is the subject of intense research. TiO_2_, with its excellent physical and chemical properties, is a very attractive material for the fabrication of chemical sensors. Meanwhile, the emerging technologies are focused on the fabrication of more flexible and smart systems for precise monitoring and diagnosis in real-time. The proposed cyber chemical systems in this paper are based on the integration of cyber elements with the chemical sensor devices. These systems may have a crucial effect on the environmental and industrial safety, control of carriage of dangerous goods and medicine. This review highlights the recent developments on fabrication of porous TiO_2_-based chemical gas sensors for their application in cyber chemical system showing the convenience and feasibility of such a model to provide the security and to perform the diagnostics. The most of reports have demonstrated that the fabrication of doped, mixed and composite structures based on porous TiO_2_ may drastically improve its sensing performance. In addition, each component has its unique effect on the sensing properties of material.

## 1. Introduction

**Figure 1:**
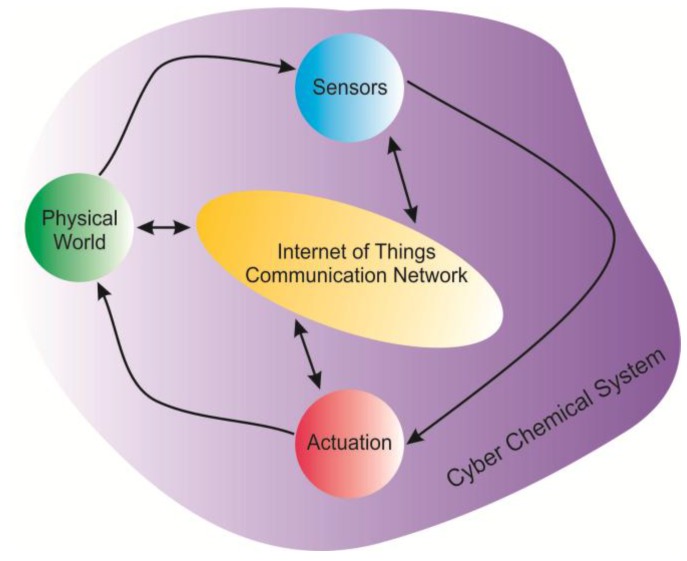
The framework of the proposed CCS.

Over the last decade the fabrication of cyber physical system (CPS) has been considered to be very promising strategy for industrial applications, smart grids and communication technology systems [1,2,3,4]. In this respect the cyber chemical system (CCS) may become a new key innovation that opens big prospects to the world of security and healthcare. The CPS is based on integration of computational applications with devices that are able to detect or control the physical changes, such as the temperature, pressure and mechanical movements [2,3]. The CCS an integration of computational applications with gas sensors for the detection of chemical changes in the environment like the presence of gaseous compounds and the variations of their concentration. The framework of CCS is depicted in Figure 1, where the main components and communication tools integrated in a smart system are shown. 

Nowadays the major environmental problems are global warming and the effects of climate change that are harming food production and have potentially devastating effects [5,6]. Moreover, the possible leakage of chemical compounds and hazardous gases in the industrial sector requires modern safety systems. The high levels of air pollution from the cars and trucks, the presence of unsafe levels of ammonia, hydrogen sulfide, volatile organic compounds (VOCs) and oxides of nitrogen demonstrate the need for increased public health protection [7,8]. In recent years, the demands for clear and renewable energy sources, such as hydrogen, have increased. This is due to the limited availability of gas, oil and coal in Nature, as well as the environmental and health risks related with their exploration and refining. Hydrogen is an odorless and explosive gas. Consequently, there is a need to control the concentration and to detect the possible leakage of hydrogen during its production and usage.

The safe transport of people and goods in airports, train and bus stations is another important issue. In order to fulfill this function, it is necessary to provide smart infrastructures for all the modes of transport, road, rail and ship. X-ray imaging of luggage and mass spectrometry techniques have been used to detect the prohibited substances and explosives [9,10]. Due to their high power consumption and large dimensions these instruments can be used only in particular cases. Therefore, they are not convenient for the wide-range of applications. Instead, there is a need of portable sensing devices that are able to detect and analyze different types of trace organic vapors, biological and chemical compounds with desirable accuracy and speed.

Real-time breath analysis are one of the best methods for the early diagnosis. These analyses can be performed using mass spectrometry techniques [11]. The needed spectrometers are large and expensive systems that are being replaced with modern small-size and portable devices based on chemical sensors [12,13].

Researchers in the sensors community and computational science have actively worked on the design and fabrication of new structures using suitable and cost effective technologies for the realization of the security and healthcare systems. These activities are aimed primarily at developing the necessary software, facilities and technological procedures for the preparation of functional nanomaterials and to facilitate the automation and control of the aforementioned systems [4,14,15].

Recent achievements in semiconductor material nanotechnology provide new opportunities. Investigations have shown that the nanostructuration, variation in composition, and design of semiconductors with different shapes have a critical effect on the optimization of their functional properties [16,17]. The synthesis of novel nanostructures with targeted performance requires a high degree of scientific creativity and the ability to develop advanced technological procedures. The materials preparation procedures should be cost effective and suitable for large scale production. Metal oxide materials with their excellent physical, chemical and multifunctional properties are the most attractive materials for the fabrication of chemical sensors [18]. The low raw material cost and the production flexibility of these structures are advantageous for their application in sensing systems. The surface structure and the specific surface area play important role on the sensing properties of materials [16,19]. In this regards, well-ordered porous metal oxide structures may be very efficient for the fabrication of gas sensors [20,21]. The porosity increases the surface area of the material, enhancing its interaction with the gaseous species. In the meantime, the well-ordered structure may improve the reproducibility of its functional properties. Research efforts to improve the traditional preparation methods and to develop novel approaches for the synthesis of porous metal oxide nanostructures have opened new perspectives and created new challenges.

TiO_2_ with its unique electrochemical and electrophysical properties is a very promising material for the fabrication of chemical sensors and its integration in security systems [22,23,24,25,26]. Especially the porous TiO_2_ quasi one-dimensional structures with their superior electron transport properties and large surface area are very attractive materials in surface chemistry, where the chemical phenomena occur at the interface of two phases, including the solid–gas interfaces (adsorption and desorption reactions) [20,27]. The pore size determines the material adsorptive and desorptive properties. Moreover, the hierarchical porous TiO_2_ nanostructures have better gas sensing performance compared with dense nanoparticle aggregates [17]. The International Union of Pure and Applied Chemistry (IUPAC) guidelines define the ranges of pore size, as shown in Table 1 [28].

As the importance of porous TiO_2_-based structures in security and healthcare applications is becoming increasingly evident, it is useful to review the research studies that have been done on this topic and to present the current state of research on porous TiO_2_ through a comprehensive overview of recent publications. The achievements in communications science in the last years have opened new perspectives for the coupling of cyber elements with the chemical gas sensors. This paper presents an overview of the achievements and challenges in the context of synthesis and study of the gas sensing properties of porous TiO_2_ structures for the fabrication of CCSs for the future applications in the smart security, smart transportation and smart healthcare.

## 2. Description, Architecture and Applications of CCSs

The wireless networks revolution led to fundamental changes to the data networking and communication tools. The integration of sensors and actuators using wireless networks with the data elaboration and analytical tools may provide an interactive functionality between processes and virtual elements [2,4]. Moreover, by realizing interconnection mechanisms between the human, machine and cyber elements it will be possible to control the processes related with the data acquisition and elaboration. Thus, the objective of CCS is to produce a system based on the integration of chemical sensors and the interaction networks providing remote control of network elements over network devices. The creation of an emerging new technology platform such as the CCS may become an excellent solution for the efficient integration of chemical sensors in smart systems using advanced technological and communication tools (cyber elements).

### 2.1. CCSs for Security

**Figure 2:**
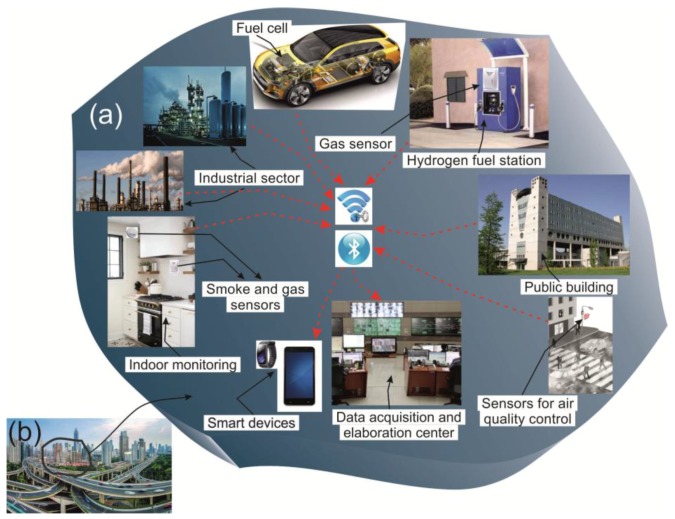
The schematic representation of the proposed CCS to improve the process safety and the quality of life. (**a**) represents an area of a smart city (**b**) based on the cyber home, cyber industry, cyber mobile and cyber society domains. The industrial sector, the hydrogen fuel stations, the streets, the hydrogen powered cars and the public buildings are equipped with the chemical gas sensors for the outdoor and indoor monitoring.

A multifunctional security model based on different CCSs is shown in Figure 2. Such a model of security activities for industrial and outdoor monitoring would improve safety and quality of life. Concerning the air quality monitoring the domestic sector is another important field, where combustible gases, CO_2_ and humidity levels have to be detected. Therefore, we should be focused on monitoring of indoor air pollution along with the outdoor pollution. The CCS for indoor monitoring is a network of gas and fire detectors connected with smart devices and data acquisition and elaboration centers. This system provides safety by monitoring public buildings and the domestic sector. Similar systems equipped with the specific gas sensors and the modern connection technologies can carry out real-time monitoring of hydrogen, methane and liquid petroleum gas (LPG)-powered cars and fuel stations to avoid explosion situations. 

**Figure 3:**
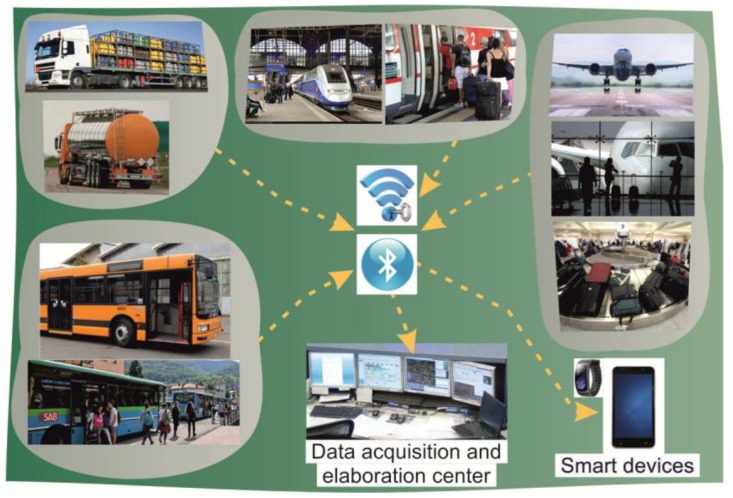
CCS applications coupling the cyber and object domain for the security of public transit and transport services. Trucks, buses, trains and train stations, airports, planes, luggage stores and luggage check instruments are all equipped with the chemical sensors.

The new safe city concept is one of a smart city equipped with a network of different types of CCSs and CPSs. The systems may operate together providing efficient security services. Figure 3 presents the application of CCSs for the security of the public transit and transport services. These devices are based on sensor arrays that screen passengers and luggage to detect organic, biological and chemical compounds. One of the main objectives of such a security system is the prevention of attacks in real-time. The integration of CCSs with CPSs may increase vehicle security by excluding the presence of illegal materials in public spaces. The application of high sensing performance chemical gas sensors in such systems can increase the security control level while reducing the involvement of human resources and decreasing the cost of monitoring.

### 2.2. CCSs for Breath Analysis and Medical Diagnostics

**Figure 4:**
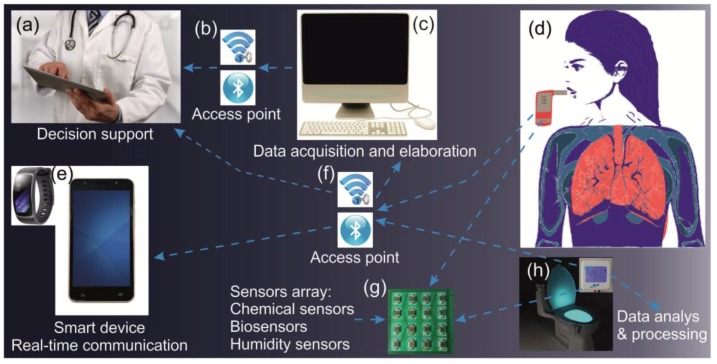
(**a**–**g**) the design and architecture of medical CCS for the breath analysis. (**h**) A smart toilet for the analysis of urine.

Non-invasive analysis is a rapidly growing field in medicine for the early diagnosis and detection of diseases. Inorganic gases (NO and CO) and VOCs (acetone, ethanol, ammonia, ethane, etc.) can be present in human breath depending on the nutrition and the cellular metabolic state in different diseases and microbial infections. The breath of lung cancer patients includes nitric oxide (NO) that causes inflammatory disorders such as asthma. Acetone is a specific breath marker for diabetes [29]. Investigations have shown that the breath acetone concentrations may be correlated with the glucose level in blood and used as a new standard for insulin management. Modern alcohol breath analyzers based on ethanol sensors may replace the traditional methods for the identification of drunk drivers. Therefore, the detection and analysis of these inorganic gases and VOCs represent valuable information sources for early diagnosis and therapy [30]. Several types of gas sensors have been examined proving the power of these innovative non-invasive approaches. Research on potential breath marker compounds for diseases and infections may provide very useful information for further medical therapy procedures [30,31]. Figure 4 shows a schematic representation of a proposed CCS for breath analysis and the smart toilet concept. The breath analysis system includes a sensor and actuator network, smart devices for real-time communication, and a data acquisition and elaboration center. The system may implement a connection between the patient and the doctor to provide medical support. The data analysis can be checked by the human using a smart device.

Another healthcare smart device is the smart toilet [32]. The smart toilet is equipped with sensor arrays to perform the analysis of urine and get the data using the network elements for data integration and elaboration (Figure 4h). The smart device displays the readings of urine tests and can provide much useful information about various diseases and conditions.

The development of aforementioned CCSs based on chemical gas sensors in future may provide the diagnostic procedures for the healthcare.

## 3. Synthesis of Porous TiO_2_ Structures

The first works on the synthesis of porous TiO_2_ structures using porous alumina templates were published about 20 years ago [33,34]. In the meantime, Zwilling et al. reported the anodic formation of TiO_2_-based tubular structures. [35]. Then, researchers started to work intensively on the development of preparation methods for TiO_2_ porous materials. Porous TiO_2_ is one of the most studied oxide materials for the wide range of applications, such as solar cells, self-cleaning systems, water splitting, gas- and biosensors, Li-ion batteries, drug delivery and implants [21,23,26,36,37,38,39]. Due to this reason, in the last a few years enormous efforts have been made to find new methods and to improve the traditional techniques for the fabrication of pure, doped, and functionalized porous TiO_2_ materials. About 4000 papers have been published on porous TiO_2_ structures during the most recent five years (source: “Web of Knowledge” database). An overview of these investigations shows that standard bottom-up approaches, such as sputtering, and physical and thermal deposition techniques have not been developed yet to obtain porous TiO_2_ structures. The preparation of porous and tubular TiO_2_ is possible through the chemical, electrochemical and template-based techniques [40,41,42].

To obtain porous and tubular TiO_2_ structures a few chemical approaches, such as the sol-gel (combined with the spin- and dip-coating methods) [40,43,44,45], evaporation-induced assembly and aerosol deposition (using chemical solutions) [46,47], have been reported. The most used and developed methods for the synthesis of porous and tubular TiO_2_ materials during the last years are atomic layer deposition (ALD), electrochemical anodization and hydrothermal synthesis. The achievements in these areas within the past few years are presented below, showing their advantages, disadvantages and perspectives for the fabrication of chemical sensors.

### 3.1. ALD

**Figure 5:**
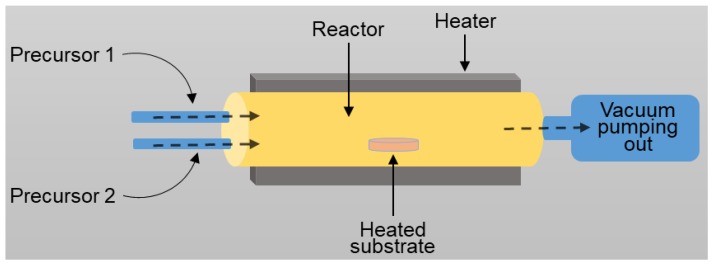
The schematics of the ALD system.

Atomic layer deposition is a template-assisted method providing material thickness control at an atomic level [42,48,49,50]. This method is relatively new compared to the other techniques for the synthesis of TiO_2_ porous materials. The deposition process is carried out in a reactor, where the precursor is pulsed and chemisorbs on the surface of a patterned substrate (Figure 5). 

Liquid, solid and gaseous precursors can be used for the ALD. The deposition temperature affects the material adhesion and growth process. The deposition is mainly performed ≥400 °C, therefore, the TiO_2_ structures obtained by ALD are mostly crystalline [50,51]. The residual reactants are removed by an inert gas purge after the deposition cycles. The most used template material for the ALD is porous alumina prepared by means of the anodic oxidation method [42,49,50,51]. The porous alumina is obtained on an appropriate substrate, followed by the porous TiO_2_ layer ALD process [52]. The TiO_2_ structures obtained on such templates have mainly tubular shape [48,49,50,52]. The length of tubes increases with the increase of the number of deposition cycles [51]. The presence of alumina under the porous TiO_2_ layer may affect its optical and electrical performance [52]. The alumina layer can be removed by selective etching [51]. Huang et al. modified the ALD technique for materials that are hard to pattern [53]. They used single crystal silicon and polysilicon layers on SiO_2_ substrates as the template material. The patterns were defined by conventional optical lithography and the samples were etched into templates using SF_6_ and radiofrequency power. Then, TiO_2_ was deposited on the high aspect ratio Si templates. In this case, the deposition temperature was relatively low (from 120 to 300 °C). Afterwards, Si cores were etched and removed. As a result, TiO_2_ nanotubes were obtained.

A few very recent studies have reported the fabrication of mixed and functionalized TiO_2_ tubular arrays by ALD [48,49]. Borbón-Nuñez et al. have used carbon nanotubes as the template. In the first step of ALD, a thin alumina buffer layer was deposited to control the thickness of material. Then, the TiO_2_ nanotubes were prepared. The presence of alumina buffer layer in the material inhibited the phase transition from anatase to rutile improving its structural stability [49]. Zhang et al. have used carbon nanocoils covered by Pt nanoclusters as the template. The amorphous TiO_2_ film was deposited by ALD on Pt/carbon nanocoil templates. The templates were removed by calcination under an air atmosphere obtaining TiO_2_/Pt porous hollow structures. Then, CoO_x_ nanoclusters were deposited onto TiO_2_/Pt producing CoO_x_ decorated TiO_2_/Pt mixture material [48].

ALD is a very precise method. It is possible to control the diameter of the tubes by varying the dimensions of the patterns. The disadvantage of ALD is the template-assisted approach that makes it difficult to use existing planar structures for the fabrication of functional devices. Besides, the deposited TiO_2_ layer is very thin. 

### 3.2. Electrochemical Anodization

**Figure 6:**
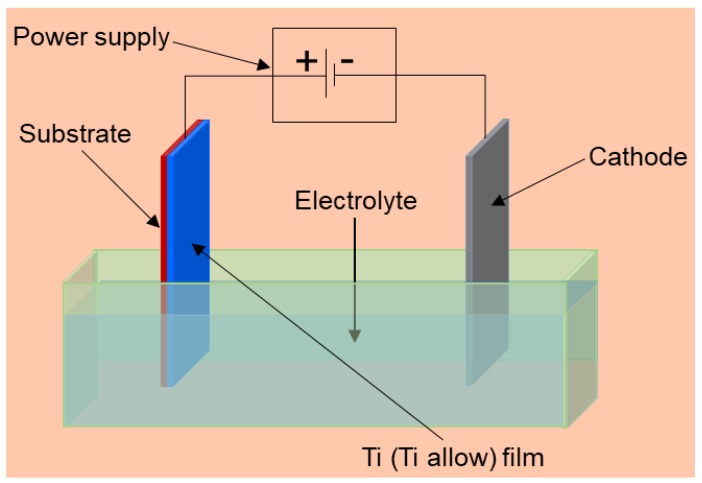
Schematic of an electrochemical anodization system.

Electrochemical anodization has been one of the traditional methods used by the industry to fabricate compact and porous films. The electrochemical formation of TiO_2_ porous arrays is based on oxidation and etching of metallic titanium layer under well-established conditions. The oxidation and etching processes are due to the presence of water and fluorine ions in the electrolyte solution. The key factor of this method is the optimization of anodization parameters for the steady state electrochemical oxidation and chemical oxide dissolution [35,54]. The anodization process is carried out in an electrochemical cell consisting of two electrodes (Figure 6). The titanium metallic layer is the anode and the other electrode is the cathode. Pt is a very stable material in different electrolyte solutions. Therefore, Pt is the most used material as a counter electrode. It is possible to obtain highly ordered and uniform TiO_2_ nanotubes using electrochemical anodization. The diameter and the length of tubes can be controlled by varying the anodization parameters, such as the electrolyte solution and temperature, the applied voltage or current, as well as the anodization time.

**Figure 7:**
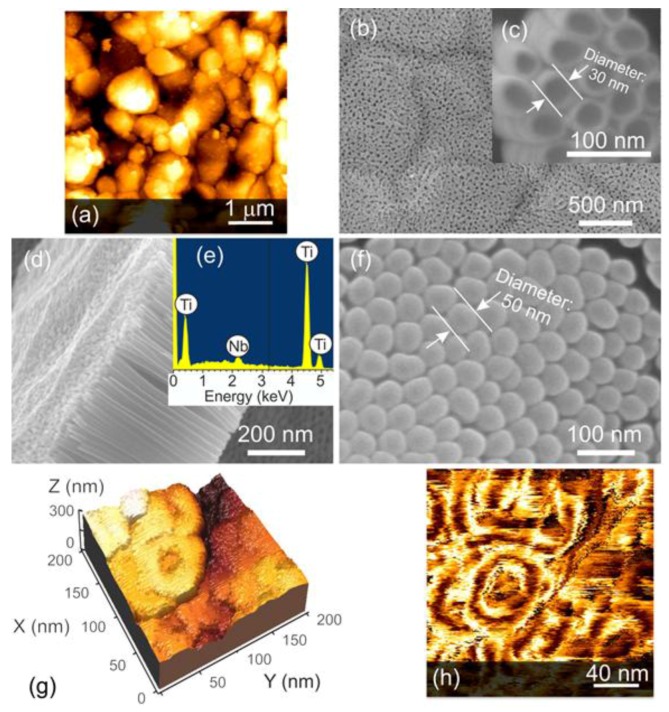
(**a**) 2D AFM topography of the alumina substrate. (**b**) Surface morphology of Nb-TiO_2_ nanotubes, (**c**) magnification of (**b**), (**d**) cross-sectional view of the anodized layer (**e**) EDX spectrum confirming the presence of 4.5 ± 0.5 wt % of Nb with respect to Ti, (**f**) the bottom-view of the tubular layer. (**g**,**h**) AFM images of the single nanotubes: (**g**) 3D topography and (**h**) the associated phase signal. Reproduced with permission from [20]. Copyright (2014) Elsevier B.V.

The research activities in recent years are mainly focused on the preparation of doped, mixed, functionalized and composite materials based on TiO_2_ nanotubes. The doping and functionalization of anodically prepared TiO_2_ nanotubes have been performed mainly by the deposition and dip coating techniques, as well as by the annealing of prepared structures in a specific atmosphere [55,56,57,58,59,60,61,62,63]. The anodic formation of doped and mixed TiO_2_ nanotubes is also possible by the anodization of titanium alloy films and by the anodization of titanium in a dopant (or mixture) material containing electrolytes [20,64]. The anodization of titanium alloys is an efficient way to obtain doped or solid solutions of TiO_2_. In this case, the metallic alloy is deposited (mainly by means of radiofrequency or direct current sputtering) on the substrate and the distribution of dopant or mixture material in the film is very homogeneous (Figure 7) [20,65]. Therefore, the anodization of such alloy materials results the growth of homogeneously mixed tubular arrays. The fabrication of doped TiO_2_ nanotubes in the dopant material containing electrolytes has been studied by Chatzitakis et al. They prepared C- and N-doped TiO_2_ nanotubes in ethylene glycol containing small amounts of water and NH_4_F. Meanwhile, they considered the content of C and N species in the electrolyte solution as the contaminants. Consequently, the concentration of dopants was not controlled during the anodization process [66].

Song et al. proposed another strategy to improve the functional properties of anodic TiO_2_ nanotubes [36]. They prepared Ti^3+^-self-doped anodic TiO_2_ nanotubes. As-prepared anodic tubes have been crystallized by thermal treatment. Then, the materials have been electrochemically reduced by controlling the reduction duration and the potential. One of the key advantages of anodization method over the other approaches is that the process is carried out by means of very facile technique without use of any vacuum apparatus. Meanwhile, as-anodized tubular arrays are mainly amorphous and a post-growth thermal treatment is needed for their crystallization [20].

### 3.3. Hydrothermal Synthesis

**Figure 8:**
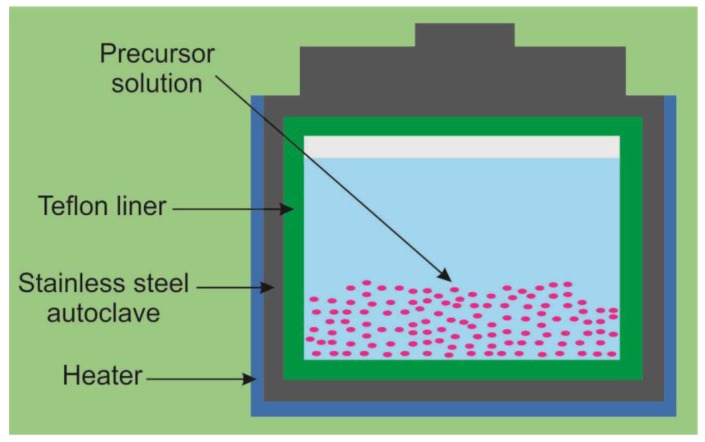
Schematic illustration of a hydrothermal growth system.

**Figure 9:**
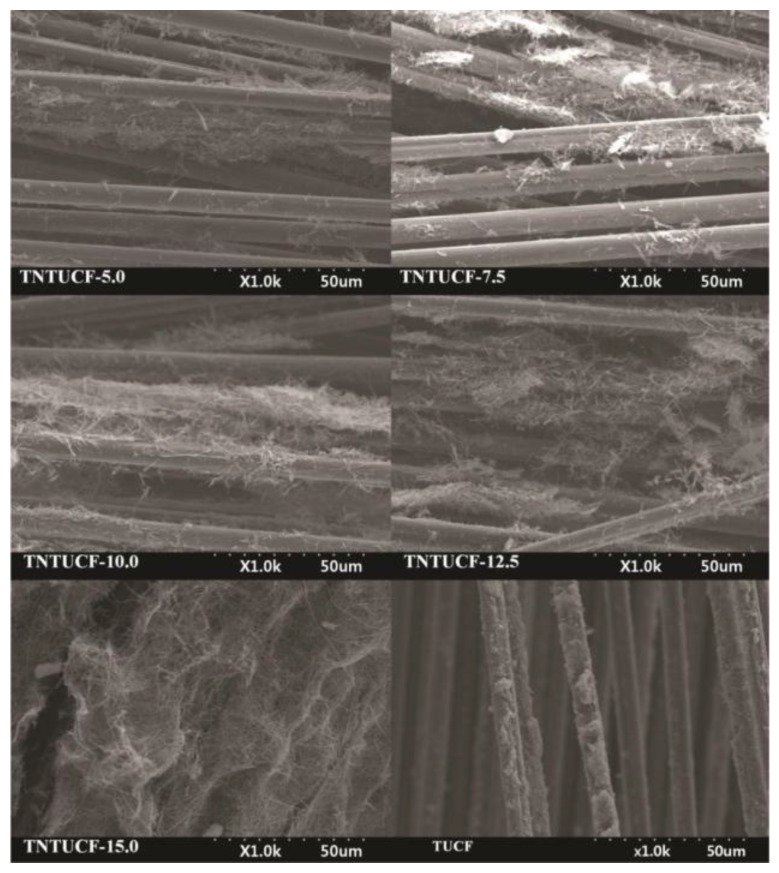
Scanning electron microscopy (SEM) of titania nanotube arrays grown on un-activated carbon fibers (TNTUCFs) with different TiO_2_ loadings (TNTUCF-5, TNTUCF-7.5, TNTUCF-10, TNTUCF-12.5, TNTUCF-15) and TiO_2_-coated UCF (TUCF). Reproduced with permission from [69].

The hydrothermal reaction is performed in aqueous solutions, where the reaction parameters, such as the solution concentration, the reaction temperature and time can be controlled. The pressure in the autoclave is also kept under control. A schematic of a hydrothermal growth system is shown in Figure 8. The hydrothermal growth process of TiO_2_ is based on the chemical reaction and solubility changes of substances in the solution at relatively high temperatures [67,68]. Since the porosity of the material plays an important role in its functional properties, in the last years the efforts have been devoted to the design of materials. A few works have reported the preparation of TiO_2_ nanotubes using templates and seed layers. Jo et al. obtained TiO_2_ nanotube arrays on carbon fibers, with the application of different TiO_2_ loadings based on the coating-hydrothermal process (Figure 9) [69]. Xie et al. used multi-walled carbon nanotube templates [70]. Luo et al. have fabricated TiO_2_ nanotubes on the porous seed layers prepared by a plasma electrolytic oxidation technique [71]. Meanwhile, the other studies have been mainly devoted to the development of synthesis parameters and the preparation of more efficient aqueous solutions for the thermal growth [72,73,74,75,76]. Ranjitha et al. investigated the effect of the reaction time on the growth of TiO_2_ nanotubes [75]. Aphairaj et al. obtained TiO_2_ tubular arrays from a natural leucoxene mineral [68]. Zhang et al. prepared porous cake-like TiO_2_ materials by the annealing of titanium-based metal-organic frameworks templates [77].

One of the main ways to modify the material surface chemistry is the doping and functionalization of the material. Information obtained from the literature indicates that the preparation of doped and mixed structures is one of the main advantages of the thermal growth technique. Researchers have reported that the doping and synthesis of solid solutions based on porous TiO_2_ can be performed by reacting two or more dispersions and solvents in an autoclave [78,79,80]. The doping and functionalization of TiO_2_ porous and tubular structures with Fe, Co, Bi_2_O_3_, Cu-Ag, CuO and graphene oxide has been successfully performed according to the aforementioned strategy [78,81,82,83,84]. Nevertheless, it is difficult to obtain well-ordered porous and tubular arrays with a homogeneous size distribution over the substrate using the hydrothermal growth technique. Besides, the growth process at relatively high temperatures limits the choice of substrates.

## 4. Fundamentals and Design of Chemical Gas Sensors

**Figure 10:**
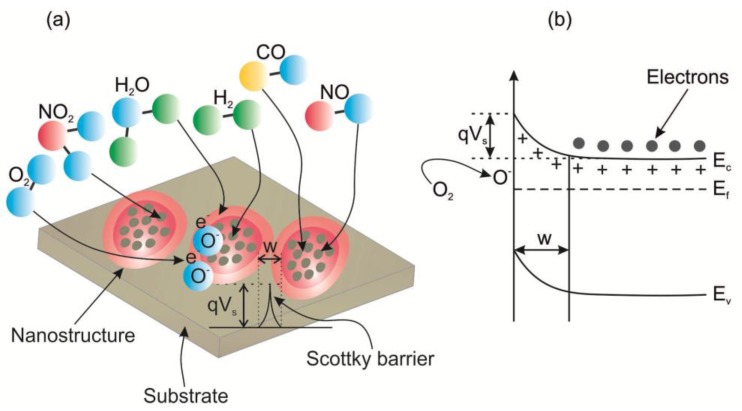
(**a**) The structural and the band model of oxide material showing the role of pores contact regions in determining the conductance over the TiO_2_ due to the adsorption/desorption process of oxidizing and reducing gases. (**b**) The model illustrating the band bending in the metal oxide material due to the ionosorption of oxygen on the material surface. E_C_, E_V_, and E_F_ denote the energy of the conduction band, valence band, and the Fermi level, respectively.

TiO_2_-based gas sensors are typically chemiresistive sensors. The working principle of a typical n-type semiconductor gas sensor material is based on its conductance change mechanism due to the adsorption/desorption process of oxidizing and reducing gases. When the material is exposed to air, oxygen is chemisorbed on its surface, extracting electrons from the conduction band and trapping them on the surface, leading to the formation of a depletion layer and a band bending (Figure 10). The height of surface potential barrier (*qV_S_*) and the depth of band banding (*W*) depend on the surface charge and can be determined according to the Equations (1) and (2), respectively, where *q* is the electrical charge of the carrier, *N_S_* is the density of surface states, *ε_r_* is the relative permittivity of the metal oxide, *ε*_0_ is the permittivity of vacuum, *V_S_* is the band bending, *n_d_* is the carrier concentration. The surface charge can be determined by the amount of ionosorbed oxygen and it depends on the Debye length (*L_D_*) (Equation (3)) [85,86]:

(1) $$qV_{S}=\frac{q^{2}N_{S}^{2}}{2\varepsilon_{r}\varepsilon_{0}n_{d}}$$

(2) $$W=\sqrt{\frac{\varepsilon_{r}\varepsilon_{0}V_{S}}{qn_{d}}}$$

(3) $$L_{D}=\sqrt{\frac{\varepsilon_{r}\varepsilon_{0}K_{B}T}{q^{2}n_{d}}}$$

*K* is the Boltzmann constant, *T* is the absolute temperature in Kelvin.

To improve the interaction between the semiconductor and the gaseous compounds the sensing material is heated. The oxygen is ionosorbed on the surface of material as molecular ($O_{2}^{-}$) and atomic species ($O^{-}$ and $O^{2-}$). This process is depends on the operating temperature of the material. The molecular form dominates below 150 °C. The atomic species dominate above 150 °C [87]. The optimal operating temperature of metal oxides varies from 200 to 500 °C [88]. Due to this reason, the oxygen is ionosorbed on the surface of sensing material mainly as $O^{-}$ ions. The $O^{2-}$ lattice oxygen is not involved in chemiresistive sensors, as its formation process occurs above 500 °C. Under ambient conditions water molecules can be adsorbed (by physisorption or hydrogen bonding) on the material surface and affect its sensing properties. Nevertheless, the studies showed that molecular water is no longer present at the material surface above 200 °C [87]. The physically absorbtion process of molecules is determined by the Van der Waals and dipole interactions [89]. A reducing gas, such as hydrogen, reacts with the chemisorbed oxygen, decreasing the amount of the surface trapped electrons according to Equation (4). Consequently, the conductance of material is increased. In the presence of other oxidizing gases, such as NO_2_ additional electrons can be extracted from the material, increasing the height of the potential barrier and decreasing the conductance (Equations (5) and (6)). These changes in conductance due to the adsorption/desorption of oxidizing and reducing gases are used as a signal for the chemical sensor devices. The surface area to volume ratio, pore size, and electron transport ability of TiO_2_ all play important roles in its functional applications [90]. For chemiresistive gas sensors, a large surface area means the presence of more surface active sites for gas absorption, resulting in noticeable material conductance changes. Therefore, the material sensing performance can be improved depending on its morphological parameters. Meanwhile, the electron exchange process is related to the *L_D_* of the material. Consequently, to enhance the conductance change the *L_D_* should be equivalent to the structure dimensions.

(4) $$H_{2}+O^{-}{}_{\left(\mathrm{ads}\right)}\to H_{2}O+e^{-}$$

(5) $${\mathrm{NO}}_{2}+e^{-}\to {\mathrm{NO}}_{2}{}^{-}$$

(6) $${\mathrm{NO}}_{2}^{-}+O^{-}+{2e}^{-}\to \mathrm{NO}+{2O}^{2-}$$

The described surface sensing mechanism is dominant when the conductivity is strongly affected by the charged adsorbate-induced band bending. TiO_2_ is a model oxide compound having three polymorphs of different symmetries, named rutile, anatase, and brookite [91]. The conductivity of rutile TiO_2_ is more affected by the bulk reduction and oxidation. It was demonstrated that the incorporation of certain dopants may affect the sensing mechanism of TiO_2_. Thus, the sensor kinetics of TiO_2_ can be described in terms of the surface chemisorption or bulk diffusion model. In particular, when Nb- and Cr-doped TiO_2_ have been tested towards oxygen at relatively high operating temperatures (~900–1050 °C), the obtained sensor device was considered as a bulk sensor. On the contrary, at the relatively low operating temperatures (≤650 °C) TiO_2_ sensors operate based on the reactions between the gaseous compounds and the preadsorbed oxygen on the surface of material. In this case, the sensing mechanism is described based on the surface chemisorption model [92].

Despite having the same chemical composition, the differences in the coordination environments, and hence chemical bonding, of rutile- and anatase-crystallized TiO_2_ result in very different ionization potentials and electron affinities. The electron affinity of anatase is higher than that of rutile and in the anatase/rutile mixed-phase TiO_2_ the generated conduction electrons flow from rutile to anatase [22]. This feature may facilitate the oxygen preadsorption on the material surface improving its response towards reducing gases [93].

Even if the theory of sensing mechanism for the semiconductor materials has been discussed and presented in several papers, it continues to be developed. Recently, Barsan et al. presented a model, where for the metal oxide based gas sensors in normal operation conditions it is possible to switch between conduction mechanisms. In the presence of humidity and at certain concentrations of a reducing gas the surface depletion layers are replaced by the surface accumulation layers and the conduction mechanism changes accordingly [94]. Hence, the precise control of the gas concentration and the concentration of relative humidity during the gas tests of a sensor device is very important.

**Figure 11:**
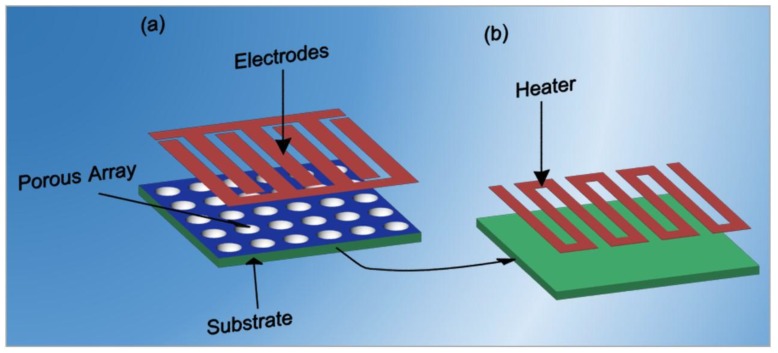
The design of a chemiresistive transducer. (**a**) The top-view of transducer: The porous structure and the interdigitated electrodes obtained on the porous array to read-out the signal. (**b**) The bottom-view of transducer with the heater deposited on the backside of substrate.

Figure 11 presents the design of a typical chemiresistive transducer, where the porous structure is obtained on the substrate, and the electrodes for the electrical measurements and a heater are deposited on the surface and the backsides of the substrate. There are different approaches to improve the architecture and the performance of sensor devices. An important topic is the substrate choice, as it can affect the power consumption of the final device. The low power consumption of a sensor makes it possible to use battery-powered instruments and plastic housings. Novel sensor devices are mainly based on thin-film technology. Therefore, the substrate material should be easily handled in the electronic industry and in the thin-film technology with the standard processes. To decrease the size, the cost and the power consumption of sensor devices it is necessary to reduce the dimensions of the substrate. The aforementioned aspects should be considered when comparing the cost and benefits of different substrates. Silicon and alumina are the materials mainly used as substrates. Silicon is the main substrate used for integrated circuits and in the planar process. Alumina substrates have well-balanced properties of insulation, thermal conductivity and breaking strength. They are available in different surface roughness and crystalline properties and can provide excellent adhesion with the thin film and thick film metallization due to the fine particles. 

Alternative polymeric substrates have been used for the fabrication of flexible and low power consumption sensors [95,96,97]. However, the polymers are typically not resistant to degradation at the high working temperatures used. Some categories of polymers, such as polyamides and polyesters, have relatively high heat-resistance and have been successfully used in the fabrication of nanomaterials and gas sensors [23,96,97,98]. These low energy consumption substrates are promising for the construction of environmentally friendly flexible sensing devices.

The chemical reactions between the gaseous species and semiconductor followed by the charge transfer in the interfaces affect the material conductance. Consequently, the choice of electrode material is very important for gas sensor devices. Recently, the chemistry and physics of barrier formation, as well as the carrier transport model for the metal/semiconductor interface including the tunnel effect have been studied and described in details [99]. The studies have shown that the contact resistance has a significant contribution to the performance of a sensor. Thus, the high stability of the materials used is one of the most important requirements for the fabrication of electrodes. It is well-known that the contacts can degrade due to the diffusion processes at electrode/oxide interface or the electrode interaction with the atmosphere. Among the noble metals, platinum is more preferable as contact material compared to gold and palladium, because of the diffusion of gold into the sensing material and oxidation of palladium at the high operating temperatures of sensors. Platinum has low diffusivity, it does not oxidize at high operating temperatures, and it is resistant to corrosive gases [18]. The deposition of an adhesion layer is also required for the preparation of high quality ohmic electrodes [99]. The careful choice of electrode material and its fabrication procedure can prevent the rather fast degradation of the contacts.

The gas tests should be carried out in a test chamber, where the relative humidity and the concentrations of gases can be held under precise control. Unfortunately, this rule is not always followed in all the works reported in the literature, which makes it difficult to evaluate the sensor performance and to compare the sensing properties of different sensors with each other. The response (*S*) of a sensing material is considered the relative change of its conductance (or resistance) due to the adsorption/desorption processes of reducing and oxidizing gases (Equation (7)):

(7) $$S=\frac{G_{0}-G_{f}}{G_{0}}$$

where the *G*_0_ is the baseline conductance of material in air and *G_f_* is the conductance in the presence of gaseous species. The response, the response and recovery times are the main parameters to evaluate the sensing performance of a material. The calculation mechanisms of sensing parameters for the different type of semiconductor materials have been discussed in details in previous reports [17,100].

## 5. Sensing Properties of TiO_2_

The most studied oxide material for chemical sensing applications is SnO_2_ [101]. Considerable attention has also been paid to ZnO gas sensors [19]. However, SnO_2_ with its sensitivity to different gases exhibits good response to humidity [102,103]. Investigations have shown that ZnO is not a stable material towards water molecules [104]. The chemical sensors are intended to detect the gases mainly under ambient conditions, where water is constantly present. Hence, this fact requires the fabrication of sensors devices that are stable to humidity changes. TiO_2_, WO_3_, In_2_O_3_, NiO and Nb_2_O_5_ have been studied for the fabrication of chemical sensors as well [105,106,107,108]. The investigations have shown that there are significant differences in water adsorption on the surface of TiO_2_ compared with the other oxide materials [68,109]. Moreover, TiO_2_ is the most used transition metal oxide in self-cleaning systems due to its excellent catalytic activity [110,111,112,113]. This effect can be used in the fabrication of TiO_2_-based self-cleaning gas sensor devices. It has been demonstrated that even the functionalization of other oxide materials with the TiO_2_ nanoparticles can induce a photocatalytic effect onto their surface. A self-cleaning sensor was obtained based on TiO_2_ and SnO_2_ materials. The organic species remaining on the structure surface were photocatalytically degraded using a UV radiation treatment. In contrast to TiO_2_/SnO_2_ structure, the sensing response of pure SnO_2_ decreases gradually depending on the increase of detection cycles [109]. Thus, porous and tubular TiO_2_ structures are promising materials for chemical sensing. However, the studies have shown that the sensing properties of TiO_2_ need to be improved to fabricate high performance sensor devices. In the meantime, the material resistance should be decreased for the manufacturing of small-size and low power consumption sensor devices.

During the last a few years, research activities have been focused on the development of new directions and strategies for the fabrication of high performance gas sensors based on porous TiO_2_. The studies have shown that the modification of the material composition and its coupling with other structures are the most efficient methods to improve the sensing properties of porous TiO_2_. Therefore, in this section recent developments in the sensing performance of doped, mixed and composite structures based on porous TiO_2_ will be presented.

**Figure 12:**
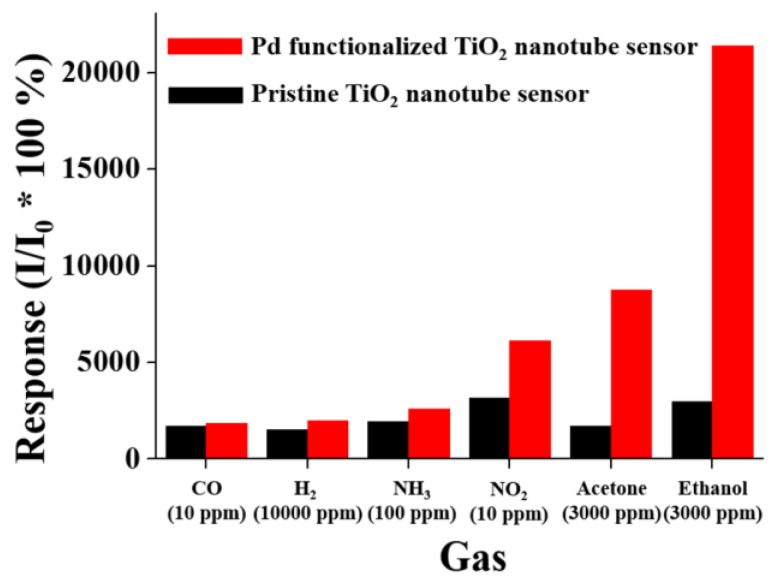
Comparison of the responses of pristine and Pd-functionalized TiO_2_ nanotubes to different gases. Reproduced with permission from [115].

Table 2 summarizes recent achievements in gas sensing applications of TiO_2_-based porous and tubular structures. It is well known that noble metals are effective catalysts for metal oxide materials. This approach has been used for the further improvement of TiO_2_ nanotubes’ sensing properties. Functionalization by Au, Pd and Pt enhances the response of TiO_2_ nanotubes towards SO_2_F_2_, ethanol, acetone and hydrogen [114,115,116,117]. The noble metals may act as active centers to catalyze the decomposition of target gas molecules and facilitate the reactions between the material surface and gaseous species. Thus, the aforementioned decomposition reactions on the material surface can enhance the sensor signal, the response and recovery times [118,119]. Park et al. improved the response and selectivity of TiO_2_ tubular structures (Figure 12) using palladium nanoparticles as the catalyst material [115]. Functionalization by Pt nanoparticles may reduce the reduction temperature of the material. In addition, the morphology, the size and concentration of Pt particles play a key role in the structure gas sensing properties [118,120]. Moreover, the noble metals are expensive and their application may increase the cost of the final devices.

Doped and solid solution sensors based on TiO_2_ porous structures have been obtained considering synergistic and catalytic effects, as well as other mechanisms for the enhancement of material sensing performance. The introduction of Ni improved the response of TiO_2_ tubular structures towards hydrogen at 200 °C by changing their response mechanism. The materials demonstrated p-type semiconducting behavior and the conductance was decreased in a reducing gas atmosphere [121,122]. The incorporation of Al and V in TiO_2_ tubular arrays also changes their response mechanism towards hydrogen. The conductance of the Al-V-TiO_2_ structure decreased upon exposure to a hydrogen-containing atmosphere [126]. Furthermore, it has been reported that the presence of Cr in the TiO_2_ lattice leads to the change of charge transfer mechanism for a high concentration of NO_2_ (50 ppm) at the elevated operating temperature (500 °C). Solid solutions based on Cr and TiO_2_ nanotubes have been obtained and treated at 400–800 °C. The materials’ conductance increased showing p-type response [123]. Pure TiO_2_ nanotubes obtained under the same conditions, showed n-type response [123,126]. The aforementioned investigations showing that at certain temperatures, depending on the gas type and concentration, the response mechanism of doped and mixed TiO_2_ nanotubes may change. Unfortunately, this response mechanism change has not been studied in details or described in the literature.

**Figure 13:**
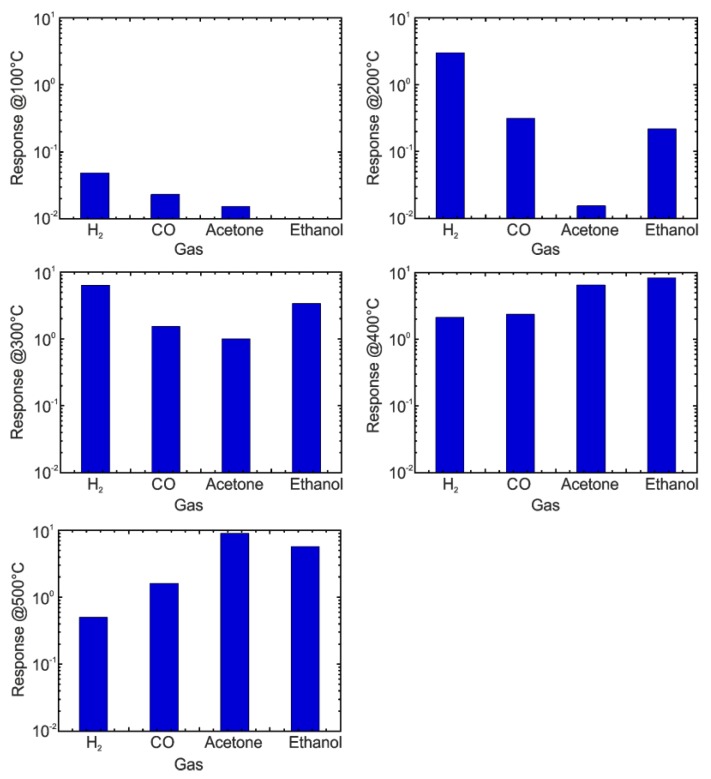
The response of niobium-containing TiO_2_ nanotubes towards 500 ppm of H_2_, 500 ppm of CO, 50 ppm of acetone and 50 ppm of ethanol at different operating temperatures (100, 200, 300, 400, 500 °C) with 40%RH @20 °C. Reproduced with permission from [20]. Copyright (2014) Elsevier B.V.

The doping and fabrication of solid solutions based on niobium and TiO_2_ nanotubes improved the sensing properties of pure TiO_2_ towards hydrogen, carbon monoxide ethanol and acetone (Figure 13). In the meantime, the niobium increased the structure conductance [20,65]. The presence of certain concentrations of Nb in the biphase (anatase/rutile) TiO_2_ inhibited anatase-to-rutile phase transition process improving the structure stability at the high working temperatures [20]. Furthermore, the preparation of biphasic TiO_2_ is beneficial to improve the material response towards hydrogen [93]. This effect can be explained by the electron transfer from rutile to anatase, which facilitates the oxygen preadsorption on the surface of the anatase structure [22,93]. Besides, anatase TiO_2_ showed better response to VOCs compared to rutile, indicating that the crystalline phase may affect the selectivity of TiO_2_ [124].

Su et al. have studied the properties of Ti^3+^ self-doped biphase porous TiO_2_. They have observed a significant decrease of material resistance due to the self-dopant Ti^3+^ [129]. The prepared structure demonstrated a selective response towards carbon monoxide at room temperature. Zhao et al. prepared MoS_2_ decorated anodic TiO_2_ tubular structures. They have obtained p-n junction of MoS_2_-TiO_2_ by means of the hydrothermal growth method. The MoS_2_-TiO_2_ composite showed a selective p-type response towards ethanol vapors [127]. Tomer et al. obtained selective ethanol sensors based on SnO_2_-TiO_2_ mesoporous hybrid materials [128]. Further Ag doping enhanced the response and recovery times of the hybrid material.

Recently, Tang et al. have demonstrated the beneficial effect of polymers on the sensing properties of TiO_2_. They coupled polypyrrole with an anodic TiO_2_ tubular structure obtaining good and selective response towards CH_2_O at room temperature. They studied the material sensing performance during the one year. Over that period the material demonstrated stabile sensing performance, as well as good stability to humidity changes [57].

**Figure 14:**
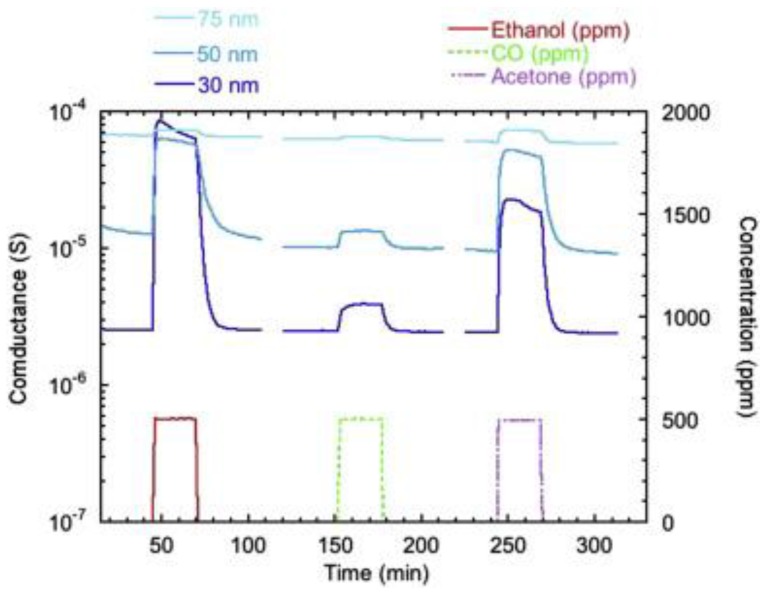
Dynamical response of Nb-doped TiO_2_ nanotubes towards 100 ppm of ethanol, carbon monoxide and acetone at a working temperature of 400 °C and 40%RH@20 °C for different internal tube diameters. Reproduced with permission from [21]. Copyright (2015) Elsevier Inc.

The incorporation of some mixed materials may increase the conductance of TiO_2_ and at the same time worsen its sensing performance or increase the structure response towards humidity. Kılınç et al. observed that the presence of high concentrations of carbon in TiO_2_ nanotubes decreases the structures’ response to hydrogen [125]. The incorporation of nitrogen increases the response of TiO_2_ to relative humidity changes [130]. The effect of humidity on the sensing performance of oxide materials should be minimized to obtain a stable sensor device. Therefore, to evaluate the effect of mixed materials on the structure sensing properties the humidity effect should be considered in parallel. The variation of pore diameter effects on the sensing performance of TiO_2_ as well. Studies have shown that a decrease in tube diameter increases the response of pure TiO_2_ tubular arrays towards VOCs (Figure 14) [21]. The porous structures of TiO_2_ studied for the sensing applications have been mainly mesoporous [20,65,114,121,124,126,128], and macroporous [57,115,116,122,123,127]. Microporous samples show responses towards carbon monoxide at room temperature. However, the self-dopant Ti^3+^ affected the sensing properties of microporous structures as well [129]. The operation of the sensing device at room temperature is favorable for the fabrication of low power consumption sensor devices. However, the sensors’ operation at such a low temperature is not advantageous for some applications. Water molecules can be formed on the surface of metal oxides due to the adsorption of some reducing gases (Equation (4)). Consequently, to provide the security in some sectors the sensor device should operate above room temperature to avoid the effects related to the presence of water.

The aforementioned investigations indicate that the fabrication of doped and hybrid structures based on the porous and tubular TiO_2_ are efficient strategies to improve the structures’ sensing performance. The response, the response and recovery times, as well as the material stability have been improved by introducing different dopants and mixed materials. Moreover, the preparation of composite structures based on tubular TiO_2_ enhances the material selectivity towards hydrogen and VOCs. The coupling of TiO_2_ with polymers may improve the selectivity and the response of materials at room temperature [57]. Another solution to overcome the selectivity issue is the fabrication of a device, a so-called electronic nose (EN), which is an array of different sensors [15,29]. We can perform cross analysis using such a device to detect the presence of a specific gas in the environment. Consequently, the CCS may get the signal not only from a single sensing device, but from two or more devices in a hybrid way, and then, provide information on a particular process based on the elaboration of data registered in a hybrid way. The fabrication of low power consumption sensors and the miniaturization of the final device are important issues for the fabrication of such CCSs. Recently, it has been demonstrated that disorder-engineered nanophase TiO_2_ (so called black TiO_2_), owing to its narrower bandgap, absorbs visible light and has relatively high electrical conductivity. These features of black TiO_2_ make the material more attractive for application in functional devices [114,115]. The decrease of TiO_2_ operating temperature and the improvement of its conductance are important for the miniaturization of the sensor devices and their integration in CCSs.

The response of porous TiO_2_ structures towards very small concentrations of acetone should be further improved since the concentration of acetone in the breath of healthy humans is usually in the range of 300–900 ppb and over 1800 ppb for the diabetic patients [131,132]. Tobaldi et al. have reported that after the functionalization of TiO_2_ nanoparticles with the Ag the material proved to be highly sensitive for the detection of acetone vapors at the low concentrations (<1 ppm) [133]. This represents an effective way to further improve the material sensing response.

## 6. Conclusions and Outlook

In this paper, an overview of the recent developments in the field of porous TiO_2_-based gas sensors and their applications in CCSs to provide safety and healthcare has been presented. To prepare highly aligned and well-ordered porous TiO_2_ structures the ALD, electrochemical anodization and hydrothermal synthesis methods have been well developed compared to the chemical approaches. ALD is a template-assisted method which allows the precise control of tube dimensions and their ordering. Meanwhile, the template-assisted approach makes the integration of the materials with the existing planar structures difficult. Anodization is a cost-effective and precise method to obtain well-ordered, doped and mixed TiO_2_ nanotubes at room temperature without the use of any vacuum technique. The anodized materials are mainly amorphous. As-anodized tubular structures are crystallized by post-growth thermal treatment. The doping and functionalization of TiO_2_ porous structures with the different materials can be successfully performed by means of the hydrothermal growth technique. In this case the material synthesis is performed at high temperatures which limits the choice of substrates.

The studies show that the preparation of composite structures based on porous TiO_2_ is a promising strategy to improve its response and selectivity. Each material in the composite affects the structures’ sensing parameters depending on the material properties, shape and amount. The introduction of dopants may improve the structure response, as well as the electrical conductance. This is an important issue for reading out the sensor signals using small size electrical devices. The presence of some dopants in the structure may improve the material conductance while worsening its sensing properties. Thus, the type and the concentration of dopant material are important issues to enhance the structure sensing performance. The fabrication of black TiO_2_ with a narrow bandgap and relatively high electrical conductivity makes the material more attractive for its applications in gas sensor systems. The improvement of TiO_2_ sensing performance for future applications in CCSs may facilitate the realization of complex systems for security uses and medical diagnoses. These systems may open new perspectives to organize smart security, smart transportation and smart healthcare.

## Figures and Tables

**Table 1:** Pore classification by the IUPAC according to the size.

Pore Width (nm)	Type of Pore
≤2	Micropores
2–50	Mesopores
>50	Macropores

**Table 2:** Gas sensing performance of TiO_2_ based porous and tubular structures at the optimal operating conditions.

Shape/Composition	TiO_2_ Crystalline Structure	Synthesis Method	Operating Temperature (°C)	Target Gas, Concentration	Response	Response/Recovery Times	Ref.
Tubular Au-TiO_2_	Anatase	Anodization	110	SO_2_F_2_, 50 ppm	(ΔR/R_0_)·100%, 19.95%	-	[114]
Tubular Pd-TiO_2_	Anatase	Anodization	200	Ethanol, 10–3000 ppm	(ΔR/R_0_)·100%, 297–21,253%	10.2/7.1 s	[115]
Tubular Pt-TiO_2_	Anatase	Anodization	150	SO_2_F_2_, 30–100 ppm	(ΔR/R_0_)·100%, ~8.65–38%	-	[116]
Tubular Ni-TiO_2_	Anatase	Anodization	200	H_2_, 1000 ppm	(ΔR/R_0_)·100%, 40%	-	[121]
Tubular Ni-TiO_2_	Anatase	Anodization	200	H_2_, 1000 ppm	(ΔR/R_0_)·100%, 13.7%	80/- s	[122]
Tubular Cr-TiO_2_	Anatase	Anodization, soaking, thermal treatment	500	NO_2_, 10–100 ppm	ΔR/R_0_, ~2–3.5	-/8–24 min	[123]
Tubular Nb-TiO_2_	Anatase, rutile	Anodization	400	Ethanol, 50 ppm	ΔG/G_0_, ~6	120/120 s	[20]
Tubular Nb-TiO_2_	Anatase	Anodization	300	Acetone, 25 ppm	ΔG/G_0_,~7	-	[65]
Tubular TiO_2_	Anatase	Anodization	200	Ethanol, 5000 ppm	(ΔG/G_0_)·100%, ~300%	-	[124]
Tubular C-TiO_2_	Anatase	Anodization, thermal treatment	100	H_2_, 5000 ppm	ΔG/G_0_, ~2	-	[125]
Tubular Al-V-TiO_2_	Anatase	Anodization	300	H_2_, 1000 ppm	(ΔR/R_0_)·100%, 50%	-	[126]
Tubular MoS_2_-TiO_2_	Anatase	Anodization, hydrothermal growth	150	Ethanol, 100 ppm	R/R_0_, 14.2	-	[127]
Porous Ag-SnO_2_-TiO_2_	Anatase TiO_2_	Chemical approaches, thermal treatment	275	Ethanol, 50 ppm	R_0_/R, ~53	3.5/7 s	[128]
Porous Ti^3+^-TiO_2_	Anatase, rutile	Chemical approaches, thermal treatment	Room temperature	CO, 100 ppm	R_0_/R, ~1.6	-	[129]
Tubular, polypyrrole based polymer-TiO_2_	-	Anodization, electropolymerization	Room temperature	CH_2_O, 1 ppm	ΔG/G_0_, 13%	-	[57]
